# Supplementary material for: ProxiNet transfers spatially learned cellular proximity to dissociated single-cell transcriptomes
Source: bioRxiv. 2026 Sep 30:2026.09.25.754372. Preprint. [Version 1] doi: 10.64898/2026.09.25.754372 (PMC13648456; doi:10.64898/2026.09.25.754372)
Supplement: Supplementary file 1 [file NIHPP2026.09.25.754372v1-supplement-1.pdf]

bioRxiv preprint doi: https://doi.org/10.64898/2026.09.25.754372; this version posted September 30, 2026. The copyright holder for this preprint (which was not certified by peer review) is the author/funder, who has granted bioRxiv a license to display the preprint in perpetuity. It is made available under a CC-BY 4.0 International license.

Supplementary Figures

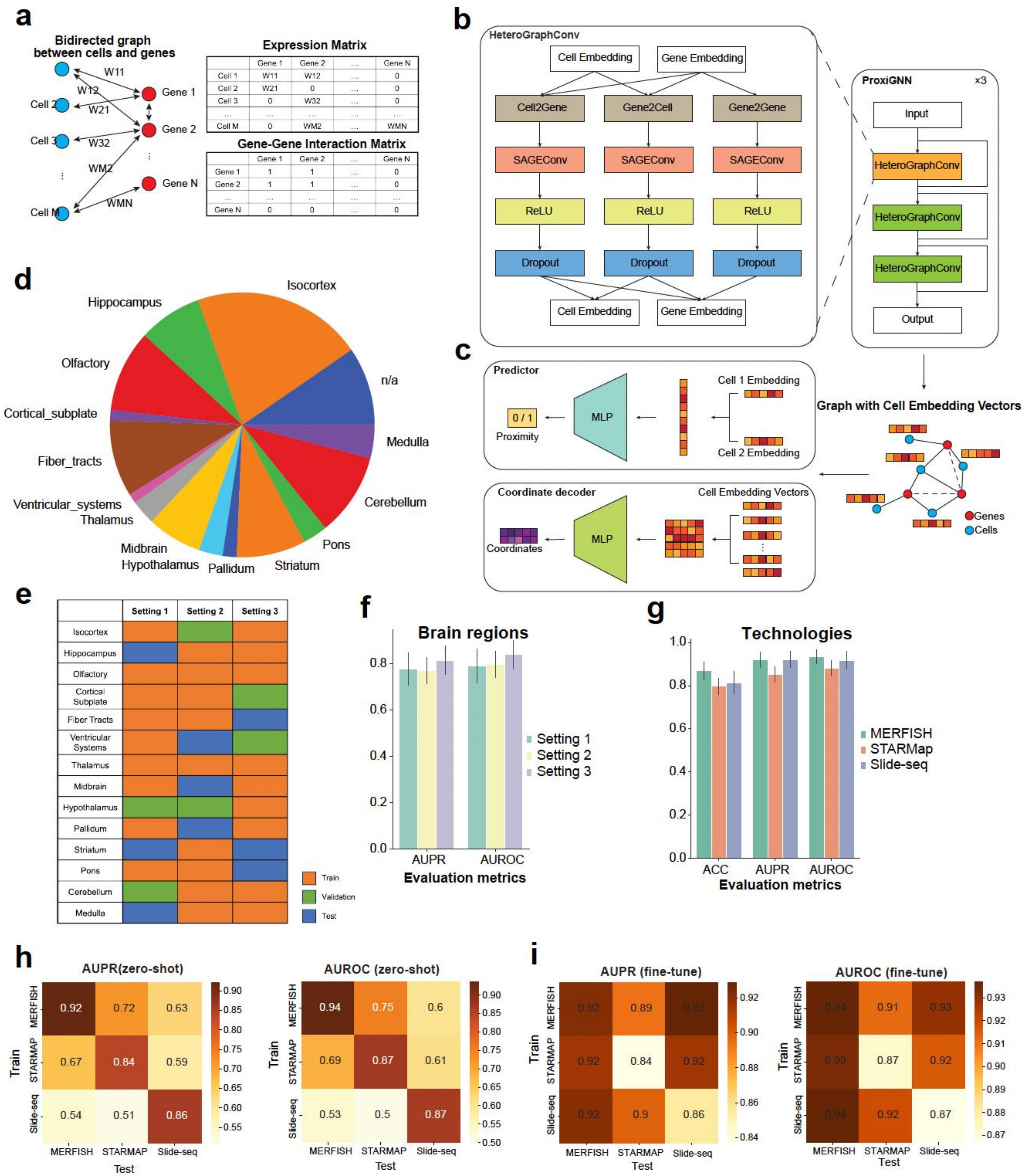

Supplementary Figure 1. Detailed ProxiNet architecture and evaluation across brain regions and spatial technologies. a, Construction of the heterogeneous cell–gene graph used by ProxiNet. The expression matrix defines weighted bidirectional edges between cell and gene nodes, and the gene–gene interaction matrix defines prior-informed edges among gene nodes. **b,** Detailed architecture of ProxiGNN. Relation-specific message passing is performed separately for Cell2Gene, Gene2Cell, and Gene2Gene relations using SAGEConv layers

bioRxiv preprint doi: https://doi.org/10.64898/2026.09.25.754372; this version posted September 30, 2026. The copyright holder for this preprint (which was not certified by peer review) is the author/funder, who has granted bioRxiv a license to display the preprint in perpetuity. It is made available under a CC-BY 4.0 International license.

followed by ReLU activation and dropout. Three sequential HeteroGraphConv layers generate updated cell and gene embeddings. **c,** Downstream use of learned cell embeddings. For the primary proximity-prediction task, embeddings from two cells are combined and passed to an MLP predictor to estimate binary cellular proximity. An auxiliary coordinate decoder maps learned cell embeddings to spatial coordinates to assess whether spatial information is retained in the learned representation. **d,** Representation of major mouse brain regions included in the spatial transcriptomic datasets used for model development and evaluation. **e,** Three brain-region train, validation, and test configurations used to evaluate generalization to held-out anatomical regions. **f,** Proximity-prediction performance across the three brain-region evaluation settings, quantified by AUPR and AUROC. **g,** Proximity-prediction performance across MERFISH, STARmap, and Slide-seq datasets, quantified by accuracy, AUPR, and AUROC. Bars show evaluation metrics and error bars indicate standard deviation across test sections. **h,** Zero-shot prediction across technology. Heat maps show AUPR and AUROC when models trained on MERFISH, STARmap, or Slide-seq are evaluated on each of the three spatial technologies. **i,** Fine-tune prediction across technology. Heat maps show AUPR and AUROC when models trained on MERFISH, STARmap, or Slide-seq are finetuned and evaluated on each of the three spatial technologies.

bioRxiv preprint doi: https://doi.org/10.64898/2026.09.25.754372; this version posted September 30, 2026. The copyright holder for this preprint (which was not certified by peer review) is the author/funder, who has granted bioRxiv a license to display the preprint in perpetuity. It is made available under a CC-BY 4.0 International license.

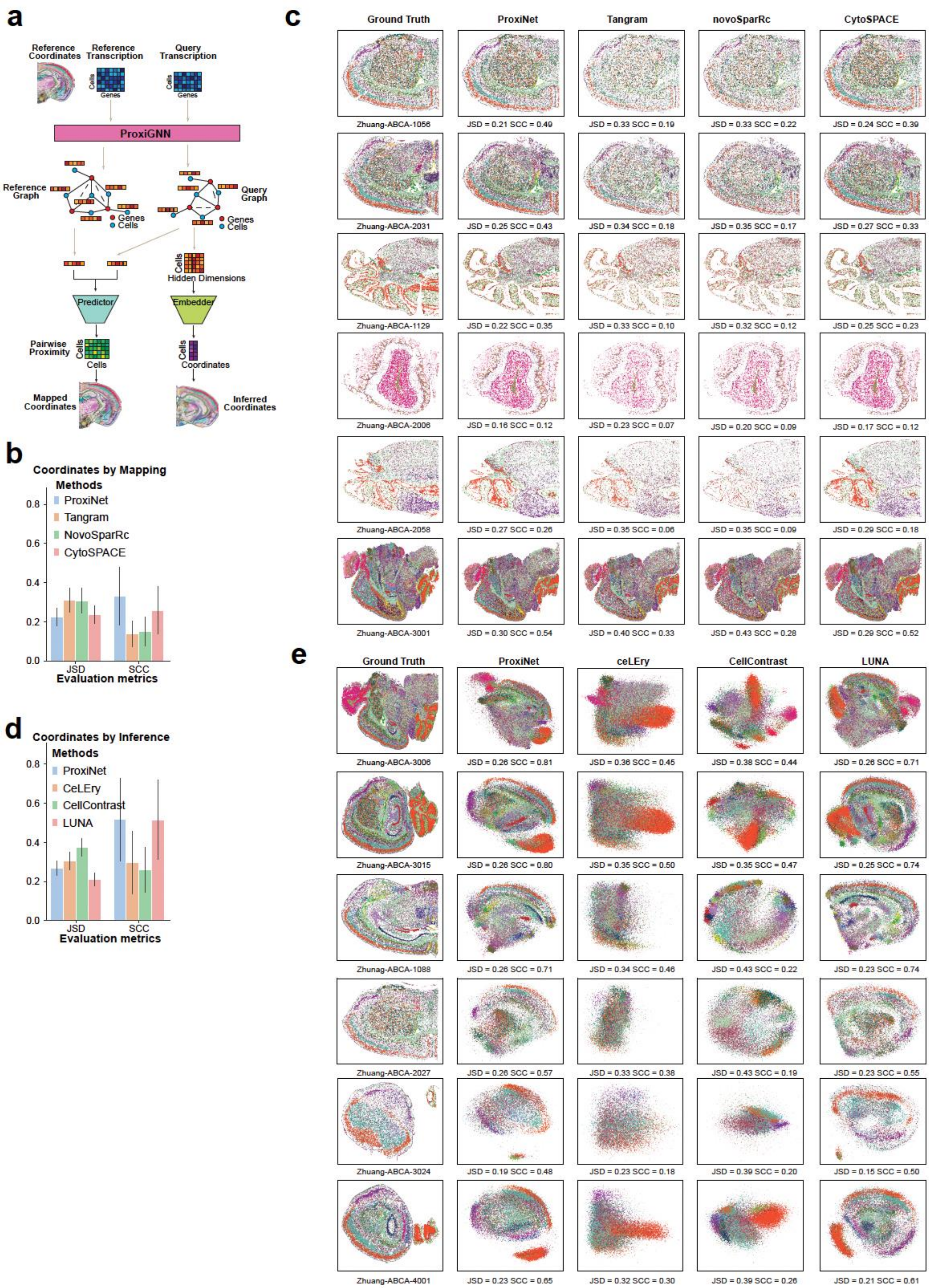

bioRxiv preprint doi: https://doi.org/10.64898/2026.09.25.754372; this version posted September 30, 2026. The copyright holder for this preprint (which was not certified by peer review) is the author/funder, who has granted bioRxiv a license to display the preprint in perpetuity. It is made available under a CC-BY 4.0 International license.

Supplementary Figure 2. ProxiNet representations retain spatial information in auxiliary coordinate-mapping and inference tasks. a, Overview of coordinate mapping and coordinate inference using ProxiNet representations. For coordinate mapping, reference spatial coordinates and transcriptomes are used together with query transcriptomes to estimate pairwise relationships and map query cells onto the reference spatial organization. For coordinate inference, learned cell representations are passed through a coordinate decoder to predict spatial coordinates directly from transcriptomic information. **b,** Quantitative comparison of coordinate mapping using ProxiNet, Tangram, NovoSpaRc, and CytoSPACE, evaluated by Jensen–Shannon divergence (JSD) and Spearman correlation coefficient (SCC). Lower JSD and higher SCC indicate greater agreement with ground-truth spatial organization. **c,** Representative coordinate-mapping results across Zhuang-ABCA-1056, Zhuang-ABCA-2031, Zhuang-ABCA-1129, Zhuang-ABCA-2006, Zhuang-ABCA-2058, and Zhuang-ABCA-3001. Ground-truth spatial organization is shown alongside results from ProxiNet and comparator methods; JSD and SCC values are indicated for each reconstruction. **d,** Quantitative comparison of coordinate inference using ProxiNet, CeLEry, CellContrast, and LUNA, evaluated by JSD and SCC. **e,** Representative coordinate-inference results across Zhuang-ABCA-3006, Zhuang-ABCA-3015, Zhuang-ABCA-1088, Zhuang-ABCA-2027, Zhuang-ABCA-3024, and Zhuang-ABCA-4001. Ground-truth spatial organization is shown alongside inferred coordinates from each method, with JSD and SCC values indicated.

bioRxiv preprint doi: https://doi.org/10.64898/2026.09.25.754372; this version posted September 30, 2026. The copyright holder for this preprint (which was not certified by peer review) is the author/funder, who has granted bioRxiv a license to display the preprint in perpetuity. It is made available under a CC-BY 4.0 International license.

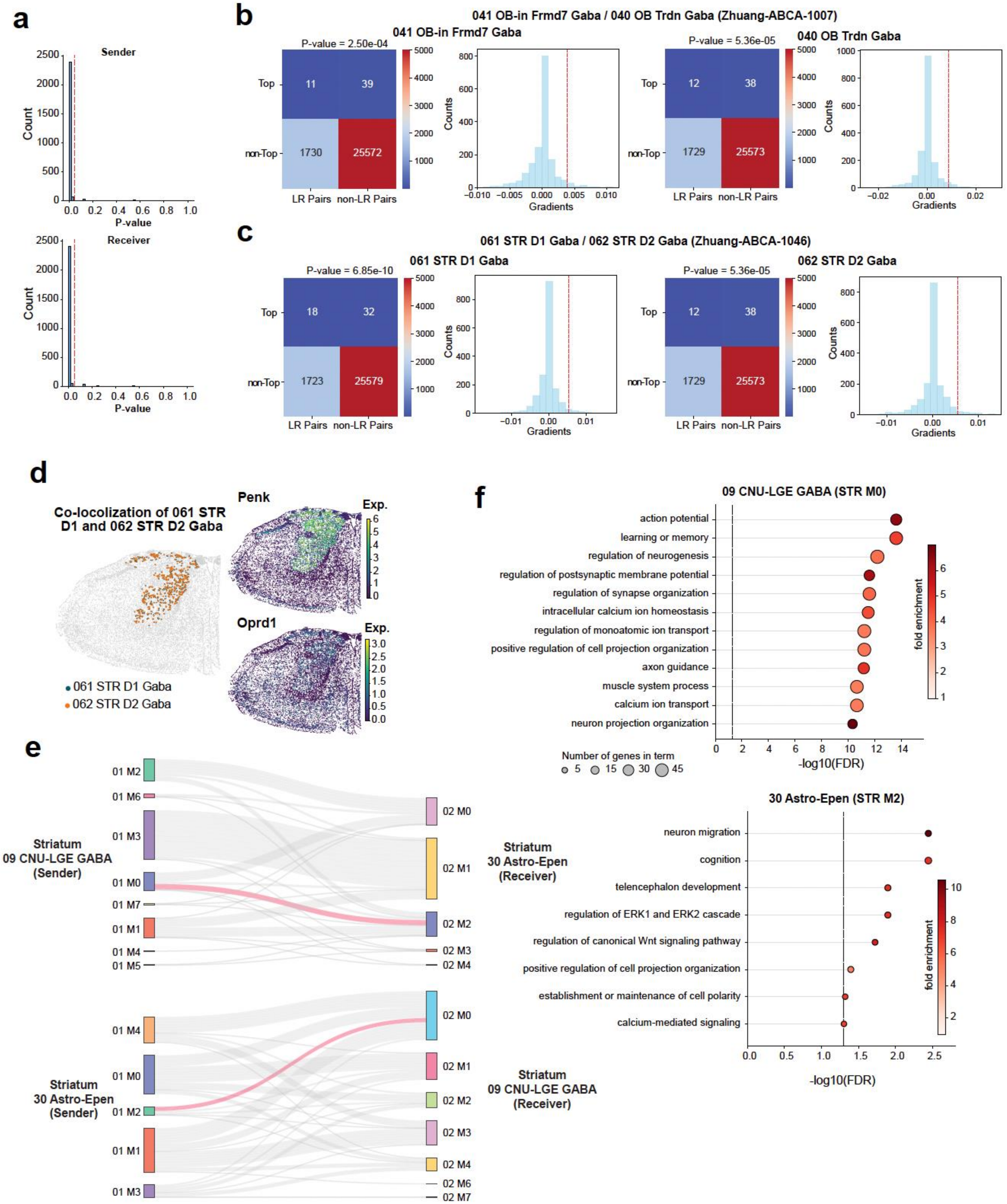

Supplementary Figure 3. Proximity-associated genes are enriched for ligand–receptor relationships and nominate broader transcriptomic programs. a, Distribution of significance values for sender- and receiver-associated genes identified by gradient-based attribution across evaluated cell-type pairs. **b,** Enrichment of ligand–receptor genes among highly attributed genes for the 041 OB-in Frmd7 GABA and 040 OB Trdn GABA

bioRxiv preprint doi: https://doi.org/10.64898/2026.09.25.754372; this version posted September 30, 2026. The copyright holder for this preprint (which was not certified by peer review) is the author/funder, who has granted bioRxiv a license to display the preprint in perpetuity. It is made available under a CC-BY 4.0 International license.

cell populations in Zhuang-ABCA-1007. Contingency tables compare ligand–receptor (LR) and non-LR genes among top versus non-top attribution-ranked genes, with enrichment P values shown above each comparison. Histograms show the corresponding distributions of gradient-based attribution scores, with dashed lines indicating the selected attribution cutoff. **c,** Equivalent ligand–receptor enrichment analysis for 061 STR D1 GABA and 062 STR D2 GABA cells in Zhuang-ABCA-1046. **d,** Spatial co-localization of STR D1 GABA and STR D2 GABA populations and spatial expression of Penk and Oprd1, providing an independent example of a ligand–receptor pair prioritized through ProxiNet attribution. **e,** Relationships between proximity-associated transcriptomic modules in Striatum 09 CNU-LGE GABA and 30 Astro-Epen cells in both sender–receiver directions. Highlighted connections denote ligand–receptor relationships prioritized between the indicated modules. **f,** Gene Ontology enrichment of representative proximity-associated modules in Striatum 09 CNU-LGE GABA (M0) and 30 Astro-Epen (M2) cells. Dot position denotes -log10FDR, dot color denotes fold enrichment, and dot size denotes the number of genes assigned to each term.

bioRxiv preprint doi: https://doi.org/10.64898/2026.09.25.754372; this version posted September 30, 2026. The copyright holder for this preprint (which was not certified by peer review) is the author/funder, who has granted bioRxiv a license to display the preprint in perpetuity. It is made available under a CC-BY 4.0 International license.

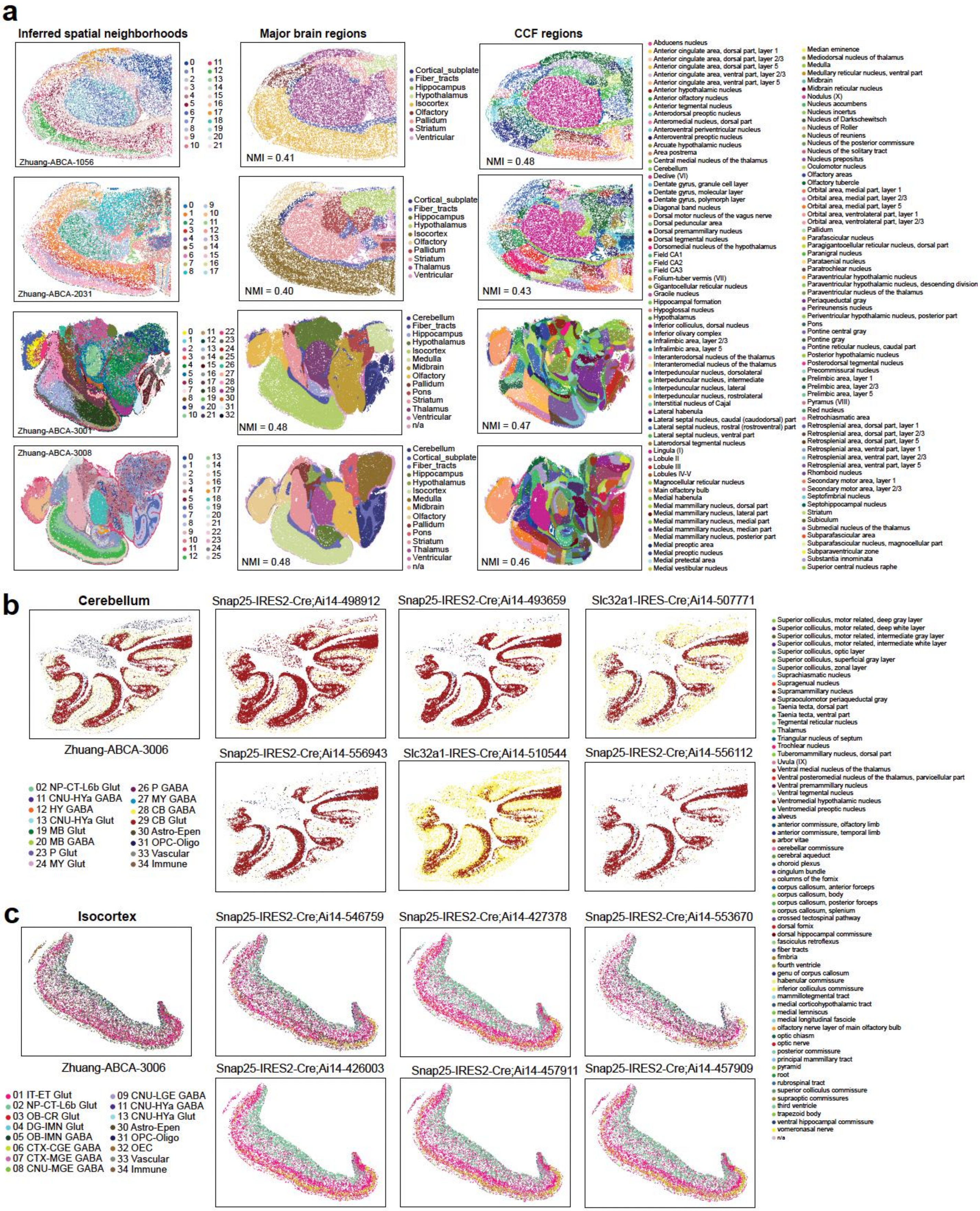

Supplementary Figure 4. Inferred cellular neighborhoods reproducibly recover anatomical tissue organization across spatial datasets. a, Expanded comparison of inferred cellular neighborhoods with anatomical organization across four spatial transcriptomic sections: Zhuang-ABCA-1056, Zhuang-ABCA-2031,

bioRxiv preprint doi: https://doi.org/10.64898/2026.09.25.754372; this version posted September 30, 2026. The copyright holder for this preprint (which was not certified by peer review) is the author/funder, who has granted bioRxiv a license to display the preprint in perpetuity. It is made available under a CC-BY 4.0 International license.

Zhuang-ABCA-3001, and Zhuang-ABCA-3008. For each section, cells are colored by inferred neighborhood assignment, annotated major brain region, or Common Coordinate Framework (CCF) region. NMI values quantify concordance between inferred neighborhoods and the corresponding anatomical annotations. **b,** Cell-type organization in the cerebellum across independent spatial transcriptomic samples. Zhuang-ABCA-3006 is shown as a reference together with multiple independent sections from the indicated mouse lines; cells are colored by major cell type to illustrate reproducibility of cerebellar cellular organization across samples. **c,** Equivalent comparison across independent Isocortex spatial transcriptomic samples, showing reproducible organization of major neuronal and non-neuronal cell populations across sections.

bioRxiv preprint doi: https://doi.org/10.64898/2026.09.25.754372; this version posted September 30, 2026. The copyright holder for this preprint (which was not certified by peer review) is the author/funder, who has granted bioRxiv a license to display the preprint in perpetuity. It is made available under a CC-BY 4.0 International license.

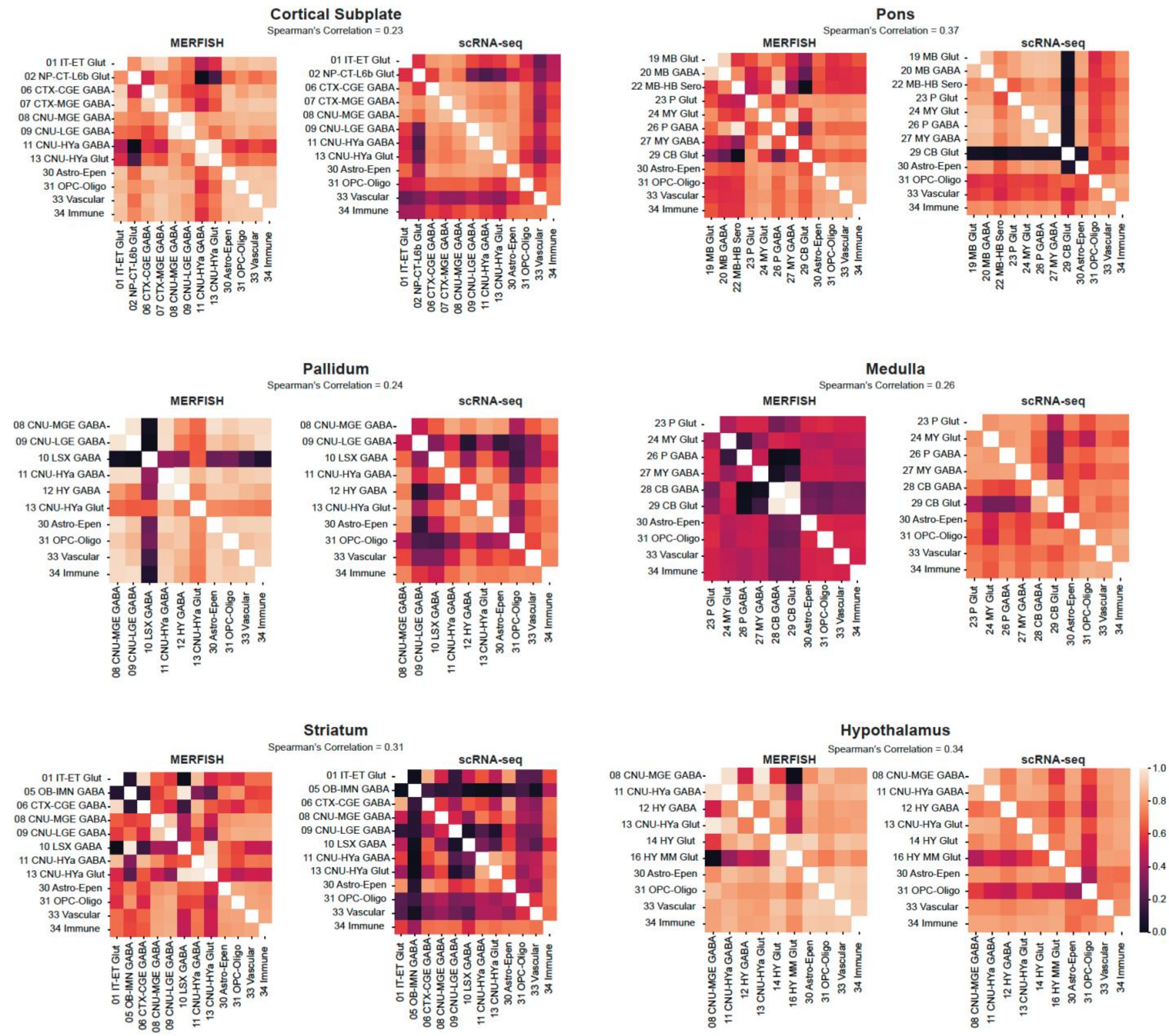

Supplementary Figure 5. ProxiNet recovers cell-type proximity relationships across multiple brain regions in dissociated scRNA-seq. Comparison of cell-type proximity relationships measured directly in MERFISH with those inferred by ProxiNet from dissociated scRNA-seq across additional brain regions. For each region, the left heat map shows MERFISH-derived cell-type proximity relationships, and the right heat map shows the corresponding relationships inferred from scRNA-seq using the spatially calibrated ProxiNet model. Concordance between the two matrices was quantified using the Spearman correlation coefficient (SCC): Cortical Subplate, SCC = 0.23; Pons, SCC = 0.37; Pallidum, SCC = 0.24; Medulla, SCC = 0.26; Striatum, SCC = 0.31; and Hypothalamus, SCC = 0.34. Together with the Cerebellum and Olfactory examples shown in Fig. 4, these analyses evaluate transfer of spatially learned cellular-proximity relationships across anatomically distinct brain regions.

bioRxiv preprint doi: https://doi.org/10.64898/2026.09.25.754372; this version posted September 30, 2026. The copyright holder for this preprint (which was not certified by peer review) is the author/funder, who has granted bioRxiv a license to display the preprint in perpetuity. It is made available under a CC-BY 4.0 International license.

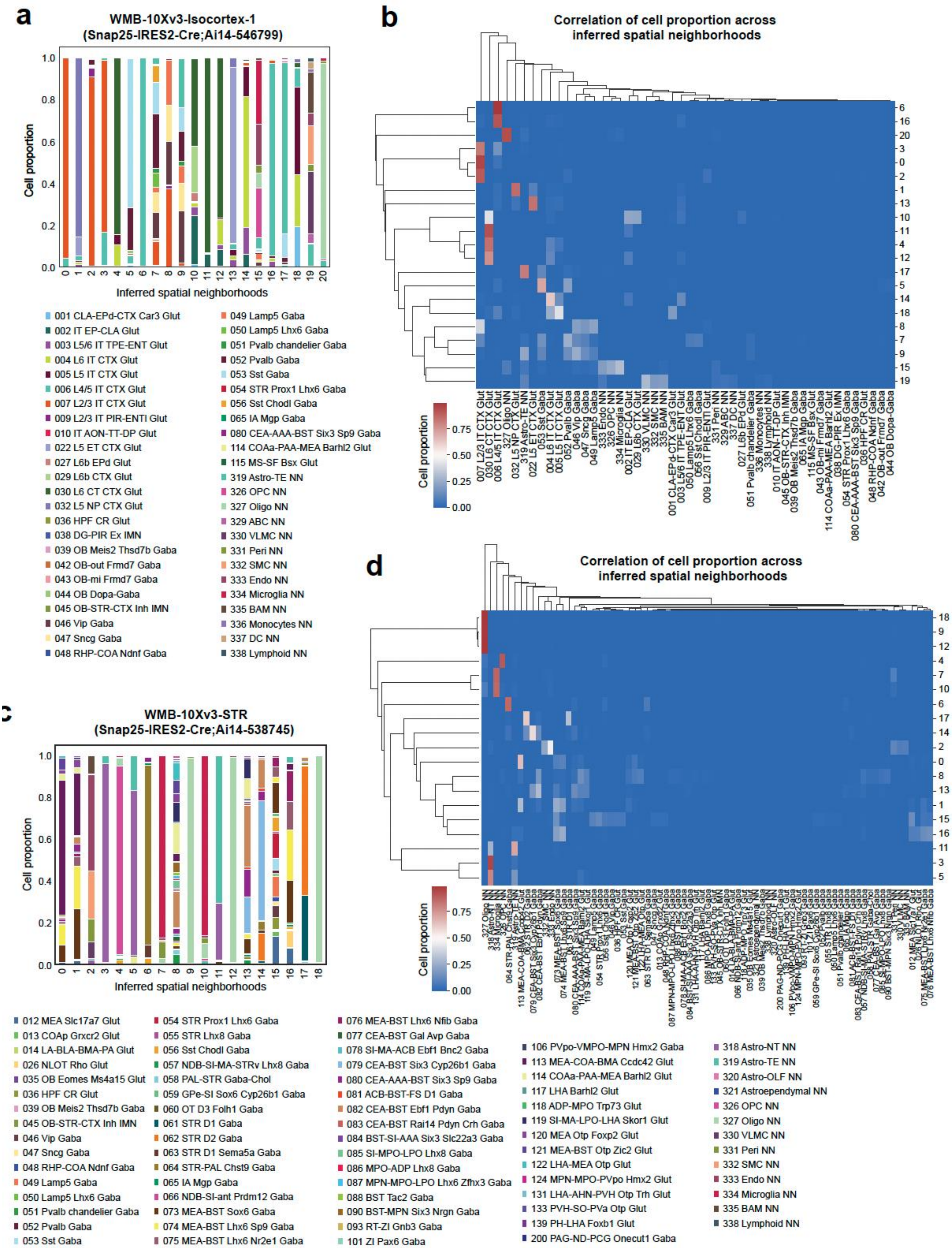

bioRxiv preprint doi: https://doi.org/10.64898/2026.09.25.754372; this version posted September 30, 2026. The copyright holder for this preprint (which was not certified by peer review) is the author/funder, who has granted bioRxiv a license to display the preprint in perpetuity. It is made available under a CC-BY 4.0 International license.

Supplementary Figure 6. Inferred cellular neighborhoods capture fine-grained cell-subclass composition in Isocortex and Striatum. a, Fine-grained cell-subclass composition of inferred cellular neighborhoods in the WMB-10Xv3-Isocortex-1 scRNA-seq sample (Snap25-IRES2-Cre;Ai14-546799). Each stacked bar represents the subclass composition of one inferred neighborhood. **b,** Hierarchically clustered representation of cell-subclass proportions across Isocortex neighborhoods. Rows correspond to inferred neighborhoods, columns correspond to annotated cell subclasses, and color denotes the proportion of each subclass within a neighborhood. Dendrograms group neighborhoods and subclasses according to similarity in composition. **c,** Fine-grained cell-subclass composition of inferred cellular neighborhoods in the WMB-10Xv3-STR scRNA-seq sample (Snap25-IRES2-Cre;Ai14-538745). **d,** Hierarchically clustered representation of cell-subclass proportions across Striatum neighborhoods, showing heterogeneous combinations of neuronal and non-neuronal subclasses across inferred cellular neighborhoods.

bioRxiv preprint doi: https://doi.org/10.64898/2026.09.25.754372; this version posted September 30, 2026. The copyright holder for this preprint (which was not certified by peer review) is the author/funder, who has granted bioRxiv a license to display the preprint in perpetuity. It is made available under a CC-BY 4.0 International license.

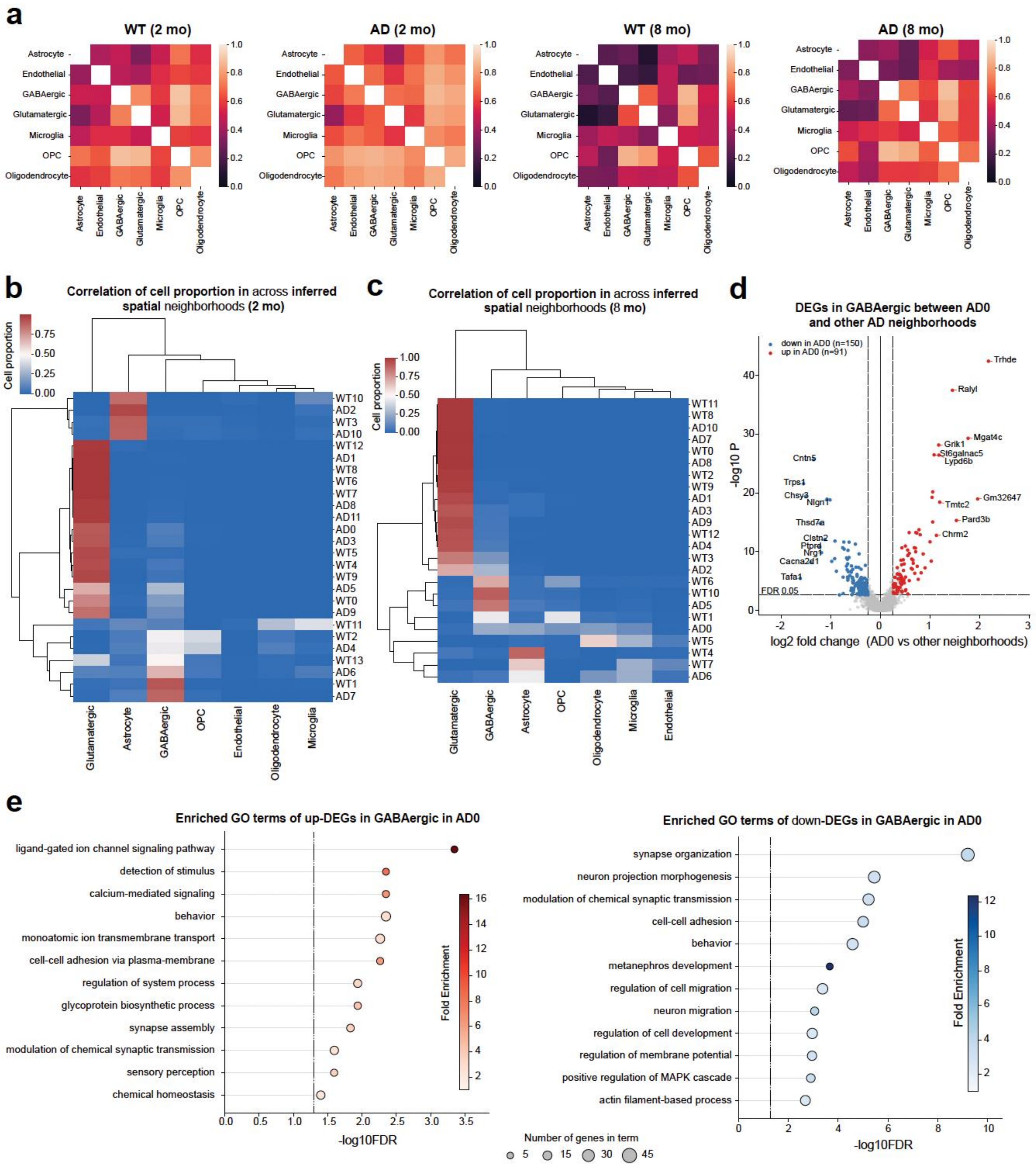

Supplementary Figure 7. Expanded characterization of age-dependent Alzheimer's-associated remodeling of inferred cellular neighborhoods. a, Predicted pairwise cell-type proximity relationships in entorhinal cortex from 2-month WT, 2-month AD, 8-month WT, and 8-month AD samples. Heat maps summarize ProxiNet-inferred proximity relationships among glutamatergic neurons, GABAergic neurons, astrocytes, oligodendrocytes, OPCs, microglia, and endothelial cells. **b,** Hierarchically clustered cell-type composition of inferred cellular neighborhoods at 2 months across WT and AD samples. Rows represent individual neighborhoods and columns represent major cell types; color denotes cell-type proportion. **c,** Equivalent analysis

bioRxiv preprint doi: https://doi.org/10.64898/2026.09.25.754372; this version posted September 30, 2026. The copyright holder for this preprint (which was not certified by peer review) is the author/funder, who has granted bioRxiv a license to display the preprint in perpetuity. It is made available under a CC-BY 4.0 International license.

of inferred cellular neighborhoods at 8 months, illustrating changes in neighborhood composition and WT–AD correspondence with disease progression. **d,** Differential gene-expression analysis of GABAergic neurons in AD0 versus GABAergic neurons from other AD neighborhoods at 8 months. The volcano plot shows log2 fold change and -log10P; 91 genes were upregulated and 150 were downregulated in AD0 at FDR < 0.05. Selected genes are labeled. **e,** Gene Ontology enrichment of genes upregulated (left) and downregulated (right) in AD0 GABAergic neurons. Dot position denotes -log10FDR, dot color denotes fold enrichment, and dot size denotes the number of genes assigned to each term.
